# 

**Published:** 1885-12

**Authors:** 


### CONTENTS.

### COMMUNICATIONS.

Pathology of Alveolar Abscess.......... 585
Alveolar Abscess and Treatment of Dead Teeth.......... 590
Hydrochlorate of Cocaine.......... 593

### PROCEEDINGS.

Illinois State Dental Society .......... 596
Ohio State Dental Society.......... 611
Virginia State Dental Association .......... 624
Report of the Ohio State Dental Examining Board .......... 626

### SELECTIONS.

Iodol.......... 627
Morphology .......... 627
SALMAGUNDI .......... 628

### EDITORIAL.

Ohio State Dental Society.......... 632
International Medical Congress.......... 633
Bibliographical .......... 636

